# Dislocation of Rotating Hinged Knee Prostheses in a Patient With Extensor Mechanism Dysfunction

**DOI:** 10.7759/cureus.24091

**Published:** 2022-04-12

**Authors:** Mohammed A Al Hassan, Omar A Salem, Emad J Alabsi

**Affiliations:** 1 Orthopedic Surgery, King Fahad Specialist Hospital, Dammam, SAU; 2 Orthopedics, King Fahad Specialist Hospital, Dammam, SAU

**Keywords:** rotating hinged knee prosthesis, total knee revision, total knee dislocation, extensor mechanism disruption, hinged knee dislocation

## Abstract

Rotating hinged knee replacements are used to restore knee stability when intrinsic stability is lost in the form of soft tissue compression. With medical engineering advancements and improvements in arthroplasty, intrinsic stability can be achieved by an implant post system. We present the case of a 44-year-old female who presented with post-traumatic right knee multi-ligamentous instability and advanced secondary osteoarthritis following a traumatic knee dislocation two years ago. The patient initially underwent a hinged total knee replacement. After five years, she got dislocation of hinged total knee replacement that affected her condition and necessitated emergency admission for open reduction and revision. Most reported cases of rotating hinge prosthesis dislocation occurred during the first year of follow-up. However, our case dislocated after five years of follow-up due to dislodgement from the tibial tray with the polyethylene channel in the form of fatigue failure of the anti-dislocation mechanism.

## Introduction

Rotating hinged knee replacements are used to restore knee stability when intrinsic stability is lost in the form of soft tissue compression. With advancements in medical engineering and improvements in arthroplasty, intrinsic stability can be achieved by an implant post system [1]. Despite the high rate of revision in these types of systems, the rotating hinged knee can be used to solve the instability issue. For the post to function properly and avoid distraction disengagement, a good soft tissue envelope needs to be adequately balanced in both flexion and extension to minimize post distraction and prevent its dislocation [2]. The amount of distraction required for implant dislocation is directly correlated with taper length, taper degree, and stem tolerance [3]. Newer designs of rotating hinged knee systems provided additional stability by longer and less tapered posts. Nevertheless, studies continue to report tibiofemoral disengagement due to the failure of the post stability mechanism. Most of such injuries are related to increased flexion laxity.

Here, we present a case of tibiofemoral disengagement with extensor mechanism dysfunction of modular rotating hinged total knee arthroplasty evaluated and treated in our institution. In addition, we share our experience with the cause of failure and the subsequent patient management.

## Case presentation

**Figure 1:**
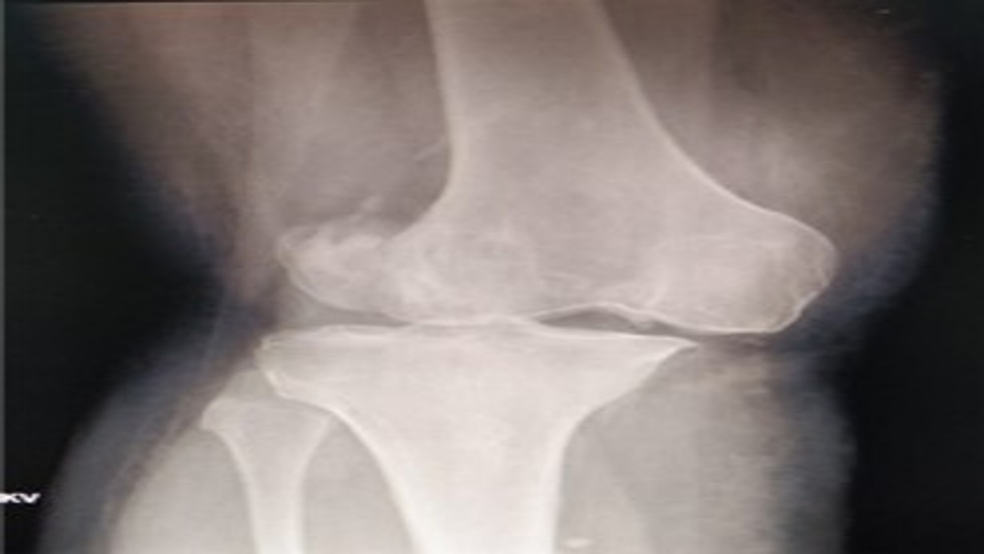
X-ray of the knee: anteroposterior view.

**Figure 2:**
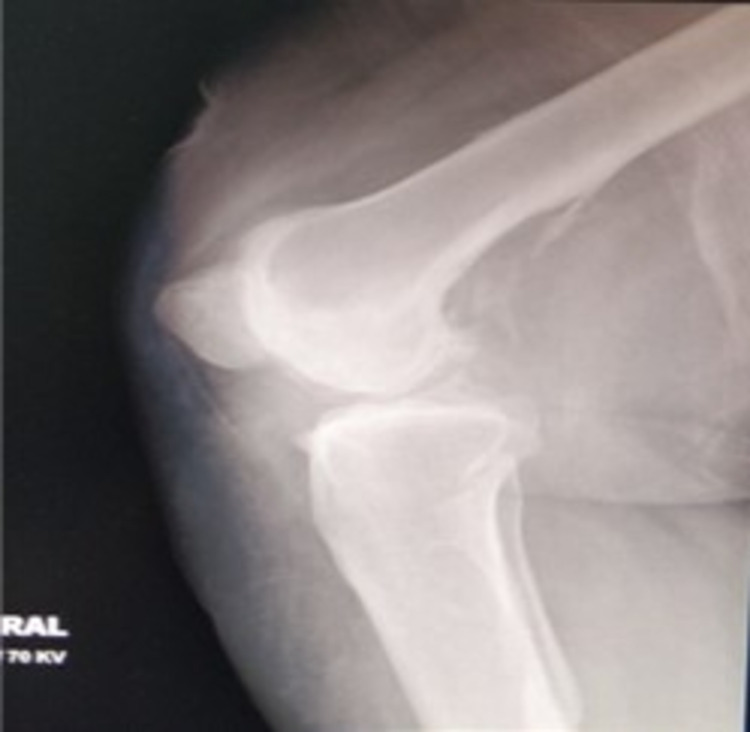
X-ray of the knee: lateral view.

**Figure 3:**
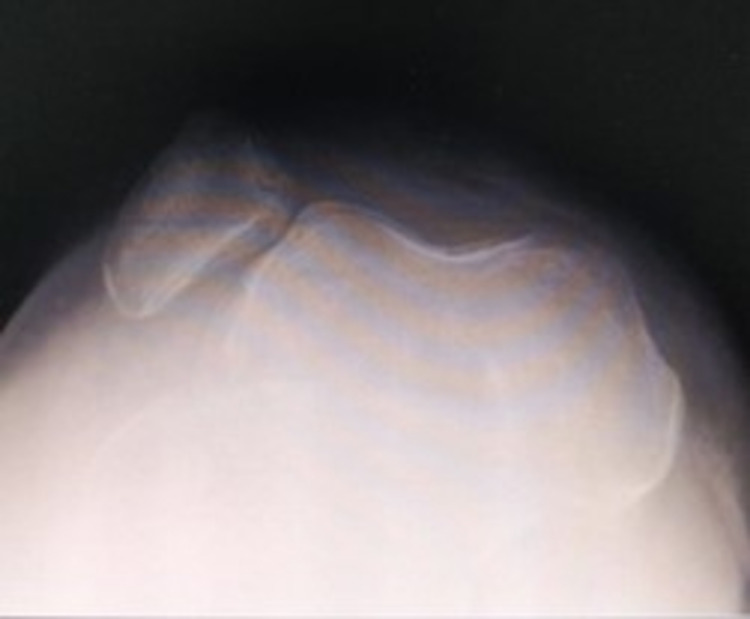
X-ray showing the axial view of the patella.

A 44-year-old female presented with post-traumatic right knee multi-ligamentous instability and advanced secondary osteoarthritis (Figures 1-3) following a traumatic knee dislocation two years ago.

Upon assessment, the patient was found to have a chronic lateral patellar dislocation. Her condition was indicated for constrained total knee arthroplasty. The patient received an RT-PLUS™ (Smith & Nephew Orthopaedics, Baar, Switzerland) modular rotating knee arthroplasty. The patella was approached during surgery by lateral retinaculum release and medial plication. Her postoperative recovery was uneventful. Upon follow-up, the patient was found to have a defective extensor mechanism. Her patella was found subluxing laterally, and plain radiographs revealed avascular necrosis of the patella. Although her activity was not optimum, it did not interfere with her daily activities, and she did not complain of pain. The patient refused further intervention to address her extensor mechanism dysfunction.

**Figure 4:**
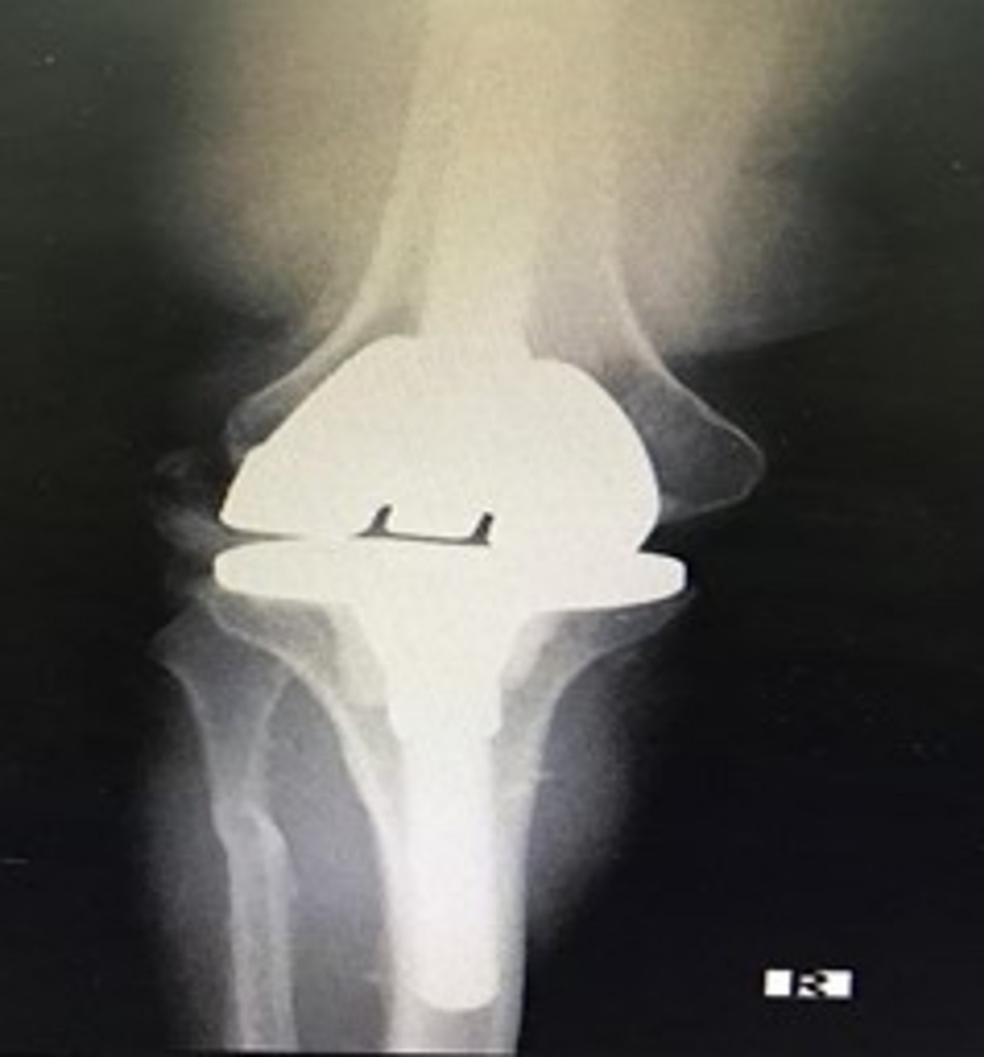
X-ray of the knee post total knee replacement: anteroposterior view.

**Figure 5:**
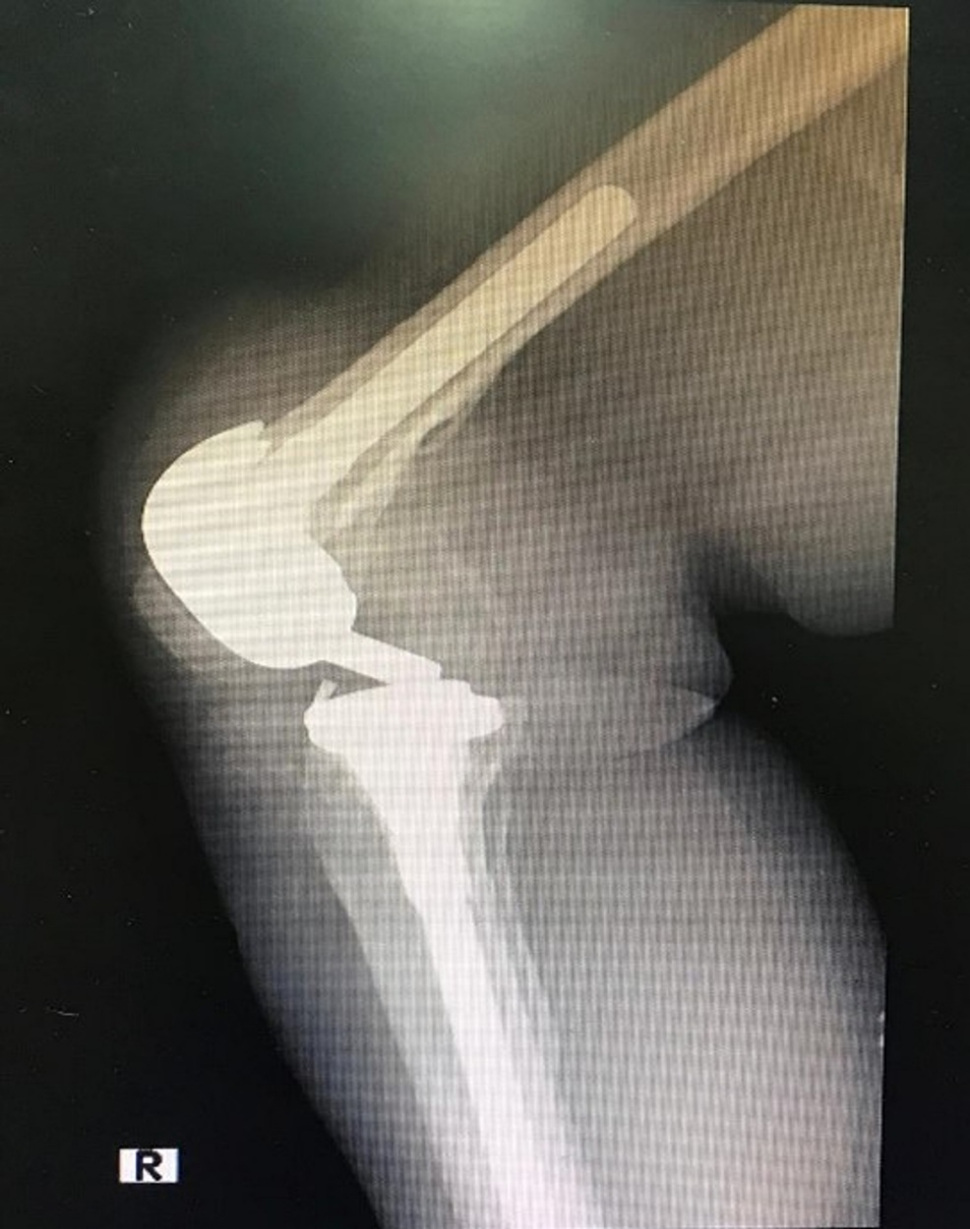
X-ray of the knee post total knee replacement: lateral view.

**Figure 6:**
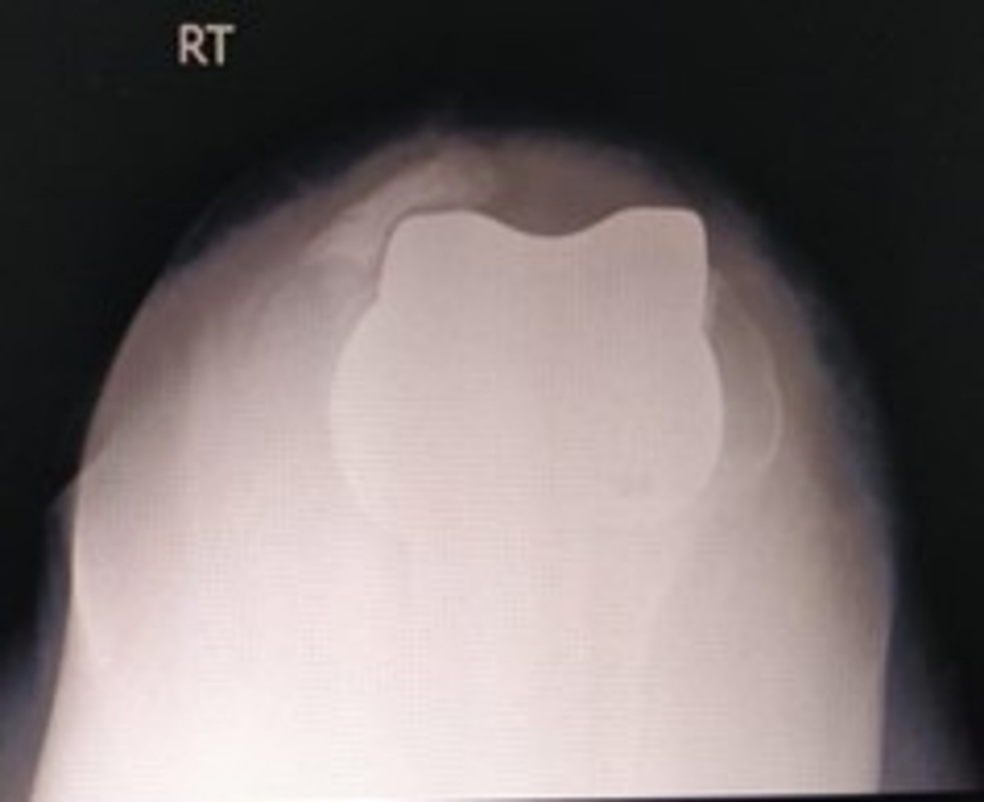
X-ray of the knee post total knee replacement showing the patella.

Five years later, the patient presented to our emergency department with right knee pain and inability to bear weight after trying to stand from a low seated position. Examination revealed a right knee deformity with her knee fixed in 30-degree flexion. Her distal neurovascular was intact. Plain radiographs (Figures 4-6) showed hinged knee dislocation with post dislodged from the canal. There were no signs of fractures or loosening. Over the past few months, the patient recalled episodes of instability while performing similar activities. This did not interfere with her daily activities. Other than that, she gave no history of trauma or pain.

**Figure 7:**
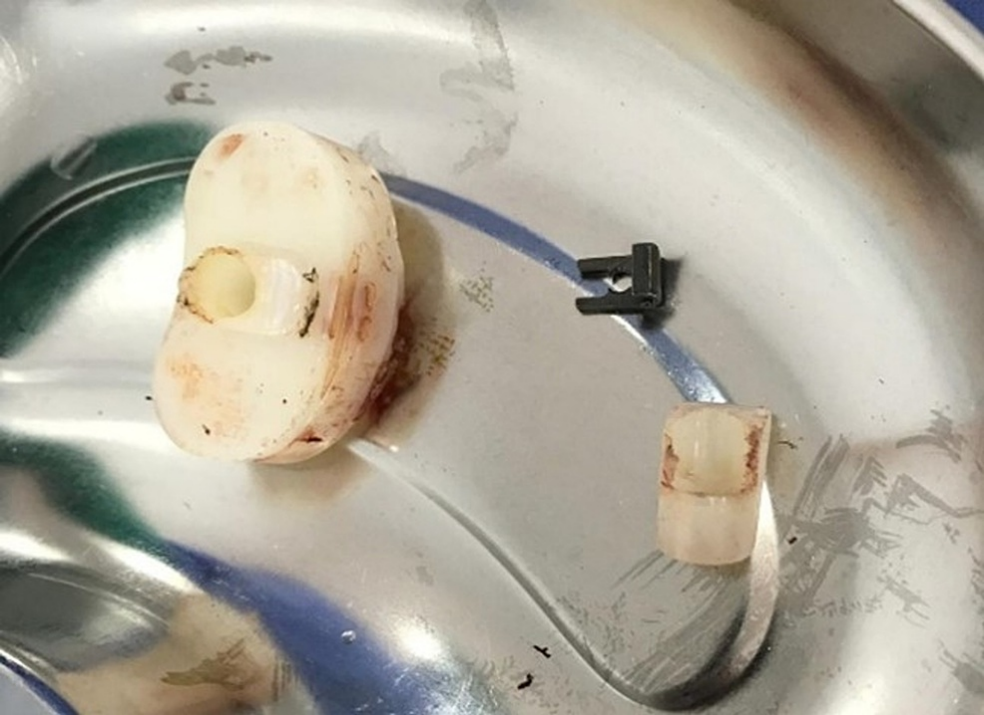
Intraoperatively, the polyethylene was dislodged from the tibial tray with the polyethylene channel broken. Signs of fretting corrosion of the post anteriorly noted.

The patient was admitted for emergency open reduction and revision. Intraoperatively, the polyethylene was found dislodged from the tibial tray (Figure 7) with the polyethylene channel broken. There were signs of fretting corrosion of the post anteriorly. The implant was found to be stable. The quadriceps and patellar tendon were intact, but the patella was dislocated laterally. The extension gap was balanced but there was a high flexion gap. Although the patella was realigned by extensive lateral release and medial augmentation, some flexion instability was felt.

## Discussion

Rotating hinged prostheses have a complication similar to any prosthesis. Dislocation is one of the complications of rotating hinge prosthesis which is mostly associated with implant breakage or distraction disengagement when patients lift or dangle their legs. There are many causes of distraction such as soft tissue instability, rheumatoid knees, trauma, and imbalance between the flexion and extension gaps [4].

Many cases of dislocation of rotating hinge knee implants have been reported [1,5]. A similar mechanism for dislocation has been reported by Ward et al. [1], Wang and Wang [6], and Pacha-Vicente et al. [7]. In our case, we also noted the same mechanism. The most important step in the mechanism of dislocation is the increment of flexion laxity that allows excessive distraction and implant dislocation. Most reported cases occurred during the first year postoperatively [1,6]. However, in this case, the dislocation occurred after five years of follow-up due to dislodgement from the tibial tray with the polyethylene channel in the form of fatigue failure of the anti-dislocation mechanism. The risk of having a dislocation in our case from our viewpoint mostly depends on the amount of laxity of the knee in flexion as a high flexion gap. We thought that, initially, the anti-dislocation feature limited the distraction and prevented dislocation; however, as seen in our case, there can be progressively loosening, permitting excessive distraction.

## Conclusions

The anti-dislocation feature in a hinged total knee replacement prosthesis gradually loosens and allows the prosthesis to get dislocated. Rotating hinge prostheses with anti-dislocation features are very useful in the treatment of knee osteoarthritis with severe bone defects and soft tissue laxity. According to this case and on comparing with other reported cases, we believe that the surgeon cannot rely solely on the intrinsic distraction stability of the total knee replacement antidislocation feature. Flexion-extension gaps must be balanced to avoid posterior capsular laxity, and the soft tissue surrounding the joint must be reconstructed. The quadriceps and patellar tendon integrity are crucial to achieve better stability.
